# Acaricidal activity of *Astragalus* polysaccharides nanoemulsion against camel tick, *Hyalomma dromedarii*

**DOI:** 10.1007/s10493-025-01085-9

**Published:** 2025-12-02

**Authors:** Shimaa Abdel-Radi, Mai A. Salem, Fady Sayed Youssef, Mohamed S. Kamel, Mohamed M. El-Bahy, Reem M. Ramadan

**Affiliations:** 1 Department of Parasitology, Faculty of Veterinary Medicine, Cairo University, Giza, 12211 Egypt; 2 Department of Pharmacology, Faculty of Veterinary Medicine, Cairo University, Giza, 12211 Egypt; 3 Department of Medicine and Infectious Diseases, Faculty of Veterinary Medicine, Cairo University, Giza, 12211 Egypt

**Keywords:** Nanoemulsion technology, Botanical acaricides, Reproductive inhibition, Integrated parasite management

## Abstract

*Hyalomma dromedarii* (*H. dromedarii*) is a major ectoparasite of camels in arid regions such as Egypt, where it causes significant economic losses and transmits several zoonotic pathogens. This study evaluated the acaricidal efficacy of a novel, locally prepared *Astragalus* polysaccharides nanoemulsified formulation (APS-NE) against various developmental stages of *H. dromedarii* infesting camels. APS-NE was prepared using ultrasonic emulsification and characterized by dynamic light scattering, transmission electron microscopy, and GC–MS, revealing uniform nanoparticle size (30–53 nm), good water solubility, and bioactive constituents including palmitic acid and quercetin derivatives. The formulation exhibited strong biological activity with very low toxicity and a wide safety margin in mice. In vitro bioassays showed a clear concentration-dependent acaricidal effect following short immersion periods: at 2% APS-NE, mortality reached 100% for eggs, larvae, nymphs, and unfed adults. Mortality rates at lower concentrations (0.5–1.5%) ranged from 63.6% to 94.6% in eggs, 33.6% to 84.3% in larvae, 53.3% to 93.6% in nymphs, and 31% to 87% in unfed adults, with larvae being the most susceptible stage. APS-NE also significantly impaired reproductive parameters in engorged females, including reductions in egg production index, egg mass, and hatchability compared to the reference drug. Strong positive correlations were observed between APS-NE concentration and tick mortality, with negative correlations for reproductive metrics. These results highlight APS-NE as a safe, eco-friendly alternative to conventional acaricides for effective *H. dromedarii* control, supporting integrated tick management strategies.

**Supplementary Information:**

The online version contains supplementary material available at 10.1007/s10493-025-01085-9.

## Introduction

Ticks are blood-feeding ectoparasites belonging to the family Ixodidae and are recognized as significant vectors for a wide range of pathogens, including viruses, bacteria, Rickettsia, and protozoan parasites that cause severe infectious diseases in both animals and humans (Muhammad et al. 2021; Ashagrie et al. 2023). Beyond their central role in disease transmission, ticks also inflict direct harm on their hosts through painful bites, which lead to skin lesions, substantial blood loss resulting in anemia, fever, and decreased productivity (Adenubi et al. 2016; Yousef et al. 2024). This issue is particularly relevant in regions where camels hold considerable economic and cultural value, such as Egypt and other Arab countries (Perveen et al. 2020). Among the various ixodid species that infest camels, the genus *Hyalomma*, especially *Hyalomma dromedarii* (*H. dromedarii*) Koch, 1844, predominates. Notably, *H. dromedarii* acts as a vector for several debilitating diseases that compromise camel health and productivity, including *Theileria camelensis*, *Theileria annulata*, *Babesia bigemina*, *Babesia caballi*, *Anaplasma* spp., and *Coxiella burnetii* (Alanazi et al. 2020; Radwan et al. 2023).

Traditionally, chemical acaricides have represented the mainstay of tick control. However, increasing evidence indicates that extensive and sometimes improper use of these agents has contributed to several critical challenges. These include the emergence of chemically resistant tick populations, environmental contamination, and the accumulation of toxic residues in animal products, such as meat and milk (Makwarela et al. 2025). As a result, there is growing interest in exploring safer and more sustainable alternatives. In this context, plant-based compounds have emerged as promising solutions due to their lower toxicity profiles and reduced potential for resistance development. Moreover, nanoparticles have garnered attention for pharmacological applications because of their enhanced bioavailability, improved pharmacokinetics, and minimized toxicity (Abdel-Rahman and Abdel-Radi 2022; Khalifa et al. 2025; Salem et al. 2025a). Thus, the integration of botanicals with nanotechnology presents a compelling avenue for developing effective biological management strategies against ticks (Radwan et al. 2023; Khater et al. 2024; Ramadan et al. 2025).

Among botanical resources, *Astragali radix*, derived from the root of *Astragalus membranaceus* Bunge is widely recognized for its medicinal properties. This herbal remedy contains an array of bioactive components, with *Astragalus* polysaccharides (APS) being the most distinctive macromolecule. APS has demonstrated a broad spectrum of biological activities, including immunomodulatory, anti-inflammatory, antidiabetic, antioxidant, and anticancer effects (Gong et al. 2018; Bakr et al. 2024). Furthermore, recent research has shown that copper nanoparticles synthesized from *Astragalus sinicus* exhibit potent acaricidal activity against *H. anatolicum* (Gattan et al. 2023). Despite these advances, there remains a lack of studies examining the acaricidal efficacy of APS-based formulations specifically against *H. dromedarii* in Egypt.

To address this knowledge gap, the present study aims to evaluate the acaricidal activity of an APS nanoemulsion (APS-NE), formulated from locally sourced APS, against *H. dromedarii* ticks infesting camels in Egypt. Our comprehensive approach involves primary characterization of APS-NE in terms of particle size and morphology and an assessment of its toxicity in a suitable animal model. Following this, we investigate its efficacy at low concentrations through a series of in vitro bioassays designed to determine dose-dependent mortality rates across different tick developmental stages after short immersion exposure. Additionally, we assess its impact on key reproductive parameters in engorged female ticks. Collectively, this research seeks to provide a sustainable and effective alternative for tick management in camel populations while contributing novel insights into the application of botanical nanoparticles in veterinary parasitology.

## Materials and methods

### Local production of APS-NE

The APS product with a purity ≥ 90.0% was sourced from Shijiazhuang ZDHF Stock-raising Co., LTD (Topvetpharm.com, Shijiazhuang City, Hebei, China). Surfactants, including Tween 80 and Tween 20, were obtained from Sigma-Aldrich Co. Deionized water was used throughout all preparation steps.

### Preparation of APS nanoemulsion (APS-NE)

The APS-NE was prepared following the method described by Fayez et al. (2025) with slight modifications. Initially, 80 mg of APS was dissolved in 20 mL of deionized water to yield an APS solution at a concentration of 4 mg/mL. For the APS group, APS was dissolved in deionized water to achieve a concentration of 580 mg/mL. To stabilize the aqueous phase, an anionic surfactant (sodium dodecyl sulfate, SDS) was added dropwise under magnetic stirring at 500 rpm for 30 min (Ferreira da Silveira et al. 2021). Massive APS was dissolved in deionized water (58 mg/mL). To stabilize the aqueous phase, sodium dodecyl sulfate (SDS, 0.5% w/v, equivalent to 50 mg for a 10 mL solution) was slowly dropped into the mixture under mechanical stirring (500 rpm) for 30 min.

For the preparation of nano-APS, APS was dissolved in the previously prepared APS solution to reach a final concentration of 58 mg/mL. The oil phase was prepared by mixing the nano-APS solution with oil at a ratio of 1:10 (v/v) relative to oil and deionized water. The oil and aqueous APS solutions were then combined and subjected to vigorous magnetic stirring at 500 rpm for 15 min to form a coarse emulsion.

This coarse emulsion was further processed using an ultrasonic probe system at 60% amplitude, employing a 1-s on/off pulse cycle. Total sonication time ranged from 5 to 15 min, optimized to achieve the desired nanoemulsion droplet size. The resultant APS nanoemulsion was centrifuged at 20,000 rpm to sediment the APS nanoparticles. The nanoparticles were washed three to four times with deionized water, then vacuum-dried overnight at 40 °C. It is noteworthy that the concentration of the active ingredient in the final nanoemulsion appeared higher than in the initial solution. This apparent increase was attributed to the removal of excess water during centrifugation, repeated washing, and vacuum drying steps, resulting in a concentrated dispersion of the active nanoparticles (up to 100 mg/mL).

### Characterization of the obtained materials

#### Dynamic light scattering (DLS) analysis

For all characterization studies, such as size distribution measurements and zeta potential analysis, the nanoparticles were dispersed only in deionized (DI) water. DI water was used as an inert, ion-free, and residue-free dispersant to eliminate the foreign ionic content affecting the surface charge and agglomeration, as well as the accuracy of the DLS and zeta potential measurements. Because DI water was the carrier solvent in all groups, no further control of the solvent group was necessary. Organic solvent (such as ethanol) could potentially disrupt nanoparticle surface chemistry and control colloidal stability, in addition to biological interactions, leading to discrepancies between measurements. Therefore, DI water was chosen as a medium for reproducibility and disturbing artifacts due to ion or organic residue leftovers(Kaur et al. 2017).

The suspension was filtered through 0.45 μm or 0.22 μm filters to eliminate large aggregates or impurities. Measurements were conducted at 25 °C with a scattering angle of 173° (backscatter). The refractive indices of both the dispersant and nanoparticles were specified according to the instrument requirements. Each measurement consisted of 3–5 runs with an equilibration time of 120 s. An appropriate analysis model (e.g., General Purpose for nanoparticles) was selected for data interpretation.

### Physical and chemical characterization

The physical and chemical properties of the APS-NE were further characterized using transmission electron microscopy (TEM). The morphology, size, and surface topography of the nanoparticles were examined using a JEOL JEM-2100 high-resolution TEM (Japan). This analysis provided detailed insights into the biological activity, shape, and surface characteristics of the APS-NE formulation.

### Gas chromatography–mass spectrometry (GC–MS) analysis

The chemical composition of the APS-NE was analyzed via GC–MS using a direct capillary column TG–5MS (30 m × 0.25 mm × 0.25 μm film thickness) on a Trace GC1310-ISQ mass spectrometer (Thermo Scientific, Austin, TX, USA) at the Regional Center for Mycology and Biotechnology (RCMB), Al-Azhar University. Prior to GC–MS analysis, APS-NE samples were derivatized to enhance the volatility and thermal stability of target compounds. Fatty acids were converted to their methyl esters (FAMEs) via acid-catalyzed transesterification. Phenolic acids and flavonoids were derivatized using standard silylation protocols. The resulting derivatives were injected directly into the GC system for separation and identification. The column oven temperature was set at 35 °C, then increased at a rate of 3 °C/min to 200 °C, followed by a final increment to 280 °C after 10 min. The injector and MS transfer lines were maintained at 250 °C and 260 °C, respectively. Helium was used as the carrier gas at a constant flow rate of 1 mL/min. A 1 μL sample was injected in split mode using the Autosampler AS1300 after a 3-min solvent delay. Electron ionization (EI) mass spectra were acquired in full scan mode at 70 eV, spanning a mass range of m/z 40–1000. The ion source temperature was set at 200 °C. Components were identified by comparing spectra with WILEY 09 and NIST 11 mass spectral libraries (Huwaimel et al. 2023).

### Determination of APS-NE cytotoxicity on vero cells

Cytotoxicity was assessed using the colorimetric MTT assay, as described by Mosmann (1983). This assay measures the reduction of yellow 3-(4,5-dimethythiazol-2-yl)−2,5-diphenyl tetrazolium bromide (MTT) to insoluble, colored (dark purple) formazan by mitochondrial succinate dehydrogenase in viable cells. After incubation, the cells were solubilized with acidic isopropanol, and the released formazan was quantified spectrophotometrically. The level of formazan production was directly correlated with cell viability.

Vero cells (1 × 10^4^ to 1 × 10⁶ cells) were seeded in 200 μL of RPMI medium supplemented with 10% fetal bovine serum (FBS) in 96-well flat-bottom plates. The plates were incubated under standard conditions at 37 °C with 5% CO₂. Following treatment with APS-based nanoemulsion (prepared using 0.1% APS at a fixed polysaccharide concentration), 20 μL of MTT solution (5 mg/mL in PBS; freshly prepared, filtered, and protected from light) was added to each well and thoroughly mixed. The plates were incubated for 4 h at 37 °C in the dark. After incubation, 200 μL of acidic isopropanol (0.1N HCl in absolute isopropanol) was added to each well, mixed, and incubated for an additional hour at 37 °C in the dark. The absorbance was measured at 570 nm (with a background correction at 630 nm) using an ELISA reader (Mosmann 1983).

### Determination of LD₅₀ of APS-NE

The median lethal dose (LD₅₀) of APS-NE was determined using the Miller and Tainter method (Miller and Tainter 1944; Okely et al. 2022). Five groups of five mice each, weighing 20–25 g, were orally administered APS-NE at doses ranging from 200 to 1000 mg/kg body weight. Toxic symptoms, mortality rates, and post-mortem findings were recorded 24 h post-administration. The LD₅₀ was calculated using the following formula:

$$\text{LD50} = \text{DM} - \left( {\sum \text{AXB}}\right)/\text{N}$$

where DM = The largest dose which kill all animals, A = Mean of dead animals between 2 successive groups, B = The constant factor between 2 successive doses, N = Number of animals in each group, Σ = The sum of (A × B).

### Testing the acaricidal effect of the prepared APS-NE

#### Collection of tick samples

Between March and December 2024, a total of 158 apparently healthy, one-humped male camels (*Camelus dromedarius*), aged 3–5 years, were examined for tick infestation prior to slaughter. These animals comprised 87 camels from Cairo slaughterhouses and 71 from those in Giza, Egypt. Hard ticks, both engorged and non-engorged (female and male), were collected from infested camels using sterile tamponed tubes. Subsequently, the collected ticks were transported to the Parasitology Laboratory at the Faculty of Veterinary Medicine, Cairo University. Morphological identification was performed according to the methods described by Okely et al. (2022), while genetic confirmation was achieved via PCR amplification of the large subunit ribosomal RNA gene locus (GenBank accession number: PV611068) (Mahdy et al. 2020; Ramadan et al. 2024a).

### Laboratory preparation of ticks for experimentation

Upon arrival at the laboratory, fully engorged, motile female ticks were individually placed into plastic containers and incubated at 25 ± 1 °C with 75–80% relative humidity (RH) in a climate-controlled incubator (Friocell, MMM, Germany) to induce oviposition. Eggs were collected daily and transferred to separate Petri dishes to ensure uniform egg age. These eggs were maintained under identical environmental conditions. To facilitate subsequent experiments, a portion of the eggs was immediately subjected to increasing concentrations of APS-NE using the egg immersion test (EIT), while the remainder was incubated until hatching into larvae.

Following hatching, the larvae were divided into two experimental groups. The first group was used to assess the acaricidal efficacy of APS-NE, whereas the second group was maintained on healthy rabbits utilizing the capsule feeding method described by Abbas et al. (2024) to obtain the nymphal stage. These larvae-fed rabbits were monitored until the development of engorged nymphs, which were subsequently divided into two groups: one exposed to varying concentrations of APS-NE, and the other incubated until molting into unfed adult ticks (Khater et al. 2016). Larvae were allowed to feed on healthy rabbits as per Abbas et al. (2024) and they were observed daily. Following engorgement and detachment, larvae were maintained under laboratory conditions (temperature, humidity, and photoperiod). Molting to the nymphal stage occurred within approximately 14 days, consistent with the literature reporting a 1–3-week molt period for ixodid ticks. Engorged nymphs were incubated at 25 ± 1 °C and 75–80% relative humidity. Under these conditions, molting to unfed adult ticks occurred within 21–28 days, consistent with previous reports for *H. dromedarii* (Perveen et al. 2020; Elgawad et al. 2023). A subset of unfed adult ticks (equal numbers of males and females) was directly exposed to APS-NE to assess their mortality. In parallel, fully engorged female ticks were incubated separately to evaluate reproductive parameters, including the egg production index, egg mass, and hatchability, following the protocols of Nabil et al. (2023) and Radwan et al. (2023). This dual approach ensured the evaluation of both lethal and sublethal (reproductive) effects of APS-NE.

### Evaluation of the acaricidal effect of APS-NE on different life stages of *H. dromedarii*

The acaricidal activity of APS-NE was systematically evaluated across different developmental stages of *H. dromedarii* using immersion techniques, as previously described by Khater et al. (2024), with minor modifications. Initially, preliminary trials were conducted to determine the appropriate concentration ranges for each tick stage. Butox 5% (deltamethrin at 1 mL/L) served as the positive control, while distilled water functioned as the negative control. Eggs, larvae, engorged nymphs, and adult ticks were individually immersed in APS-NE for 60 s under controlled environmental conditions (25 ± 1 °C, 75–80% RH). Post-treatment, ticks were rinsed three times (30 s each) with distilled water and subsequently dried on Petri dishes lined with Whatman No. 1 filter paper. The treated ticks were then incubated at 25 ± 1 °C and 75–80% RH. Mortality was recorded daily for each developmental stage, while in engorged females, oviposition and reproductive parameters (egg mass, egg production index, and hatchability) were assessed following Nabil et al. (2023) and Radwan et al. (2023).

### APS-NE concentrations and monitoring procedures

Developmental stages were exposed to incrementally increasing concentrations of APS-NE (ranging from 0.5% to 12%, depending on the stage) to identify the concentration yielding the highest mortality rate. Both exposed and control groups were observed daily under consistent environmental conditions using a binocular microscope (LEICA DM 750, Russia) and a hand lens. Mortality was recorded up to 15 days post-exposure, or until all individuals in the exposed group had died. Each concentration was tested in triplicate, and mortality percentages were calculated by comparing the number of alive and dead ticks in both experimental and control groups.

### Acaricidal effect of APS-NE on tick eggs

To specifically assess the impact of APS-NE on embryonated tick eggs (3–5 days post-oviposition), approximately 500 viable eggs were immersed for one minute in 1 mL of APS-NE at concentrations of 0.5%, 1%, 1.5%, and 2% (Ramadan et al. 2023). Following treatment, the emulsions were removed, and the eggs were incubated under standard laboratory conditions (25 ± 1 °C, 75–80% RH) in sealed plastic containers covered with muslin cloth.

### Effect of APS-NE on tick larvae

Similarly, as per the EIT protocol, 500 active, motile larvae older than one week were exposed to APS-NE at concentrations of 0.5%, 1%, 1.5%, and 2% using the larval immersion test (LIT). After exposure, the larvae were incubated at 25 ± 1 °C and 75–80% RH. Mortality was recorded daily for 15 days in both the exposed and control groups, following the protocols of Khater et al. (2016) and Radwan et al. (2023). The larvae were considered dead if they showed no reaction after gentle stimulation with a fine brush. Ticks displaying only uncoordinated or spasmodic movements (ataxia) were also recorded as dead, as described by Radwan et al. (2023).

### Effect of APS-NE on the nymphal stage of *H. dromedarii*

For the nymphal stage assessment, 180 motile, engorged nymphs were collected from experimentally infected rabbits and allocated into groups of 10 per dish. Each group was immersed in 5 mL of APS-NE at concentrations of 0.5%, 1%, 1.5%, and 2% for one minute. Additional groups were exposed to Butox 5% and distilled water as positive and negative controls, respectively. Post-immersion, engorged nymphs were washed, dried, and incubated at 25 ± 1 °C and 75–80% RH. Mortality was recorded daily for 15 days, based on the absence of motility or failure to molt into adults (Elgawad et al. 2023).

### Effect of APS-NE on adult *H. dromedarii* ticks

#### Unfed adult ticks

To evaluate the effect of APS-NE on unfed adults, 180 active, unfed ticks (equal numbers of males and females) were divided into groups of 10 per dish. Each group was immersed in APS-NE at concentrations of 1.5%, 3%, 6%, and 12%. The reference (Butox 5%) and negative (distilled water) control groups were included for comparison. After exposure, the ticks were dried, incubated under standard conditions, and monitored daily for mortality over a 7-day period (Mohammed et al. 2022).

### Engorged female ticks

The impact of APS-NE on engorged females was assessed using a modified protocol described by Khater et al. (2024). Fully engorged females were weighed and divided into groups of 15, each further subdivided into three replicates of five ticks. Each group was immersed for one minute in 10 mL of APS-NE at concentrations of 1.25%, 2.5%, 5%, and 10%. Following treatment, the ticks were rinsed, dried, and incubated under standard conditions for 15 days. During this period, egg masses were collected and incubated to determine their hatching success.

Reproductive performance was quantified by calculating the egg production index (EPI) as follows: EPI (Egg mass (mg)/Initial weight of engorged female (mg)) × 100. Additionally, the hatchability percentage of deposited eggs was recorded as described by Nabil et al. (2023).

### Statistical analyses

Statistical analyses were conducted using R and SPSS 26 software (Ramadan et al. 2024b). One-way ANOVA, followed by Fisher’s LSD test for multiple comparisons, was used to compare the differences between groups (Abdel-Radi et al. 2022; Taha et al. 2024, 2025; Salem et al. 2025b,c). For LD50 evaluation, a nonlinear regression model was applied. Spearman correlation analysis assessed the relationship between APS-NE concentrations and mortality at different life stages of *H. dromedarii*, as well as reproductive parameters of *H. dromedarii* females. A significance level of P < 0.05 was used for all analyses (Mahdy et al. 2017; Salem et al. 2024).

## Results

### Nanoparticle characterization

**Fig. 1:**
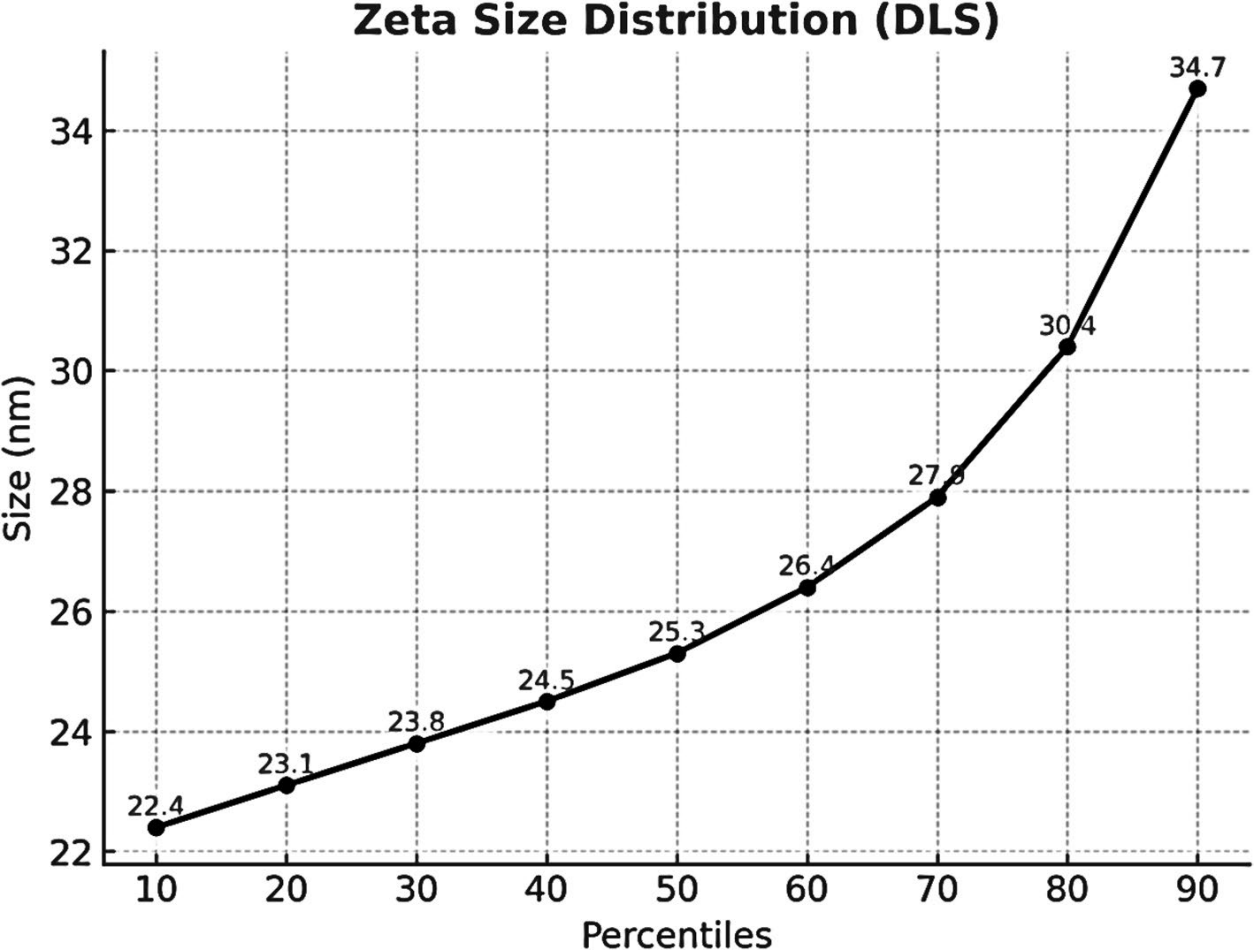
The number-based dynamic light scattering (DLS) percentile size distribution of the nanoparticles shows D10 = 22.4 nm, D50 = 25.3 nm, and D90 = 34.7 nm. most of the particles lie within the 20–30 nm range

**Fig. 2:**
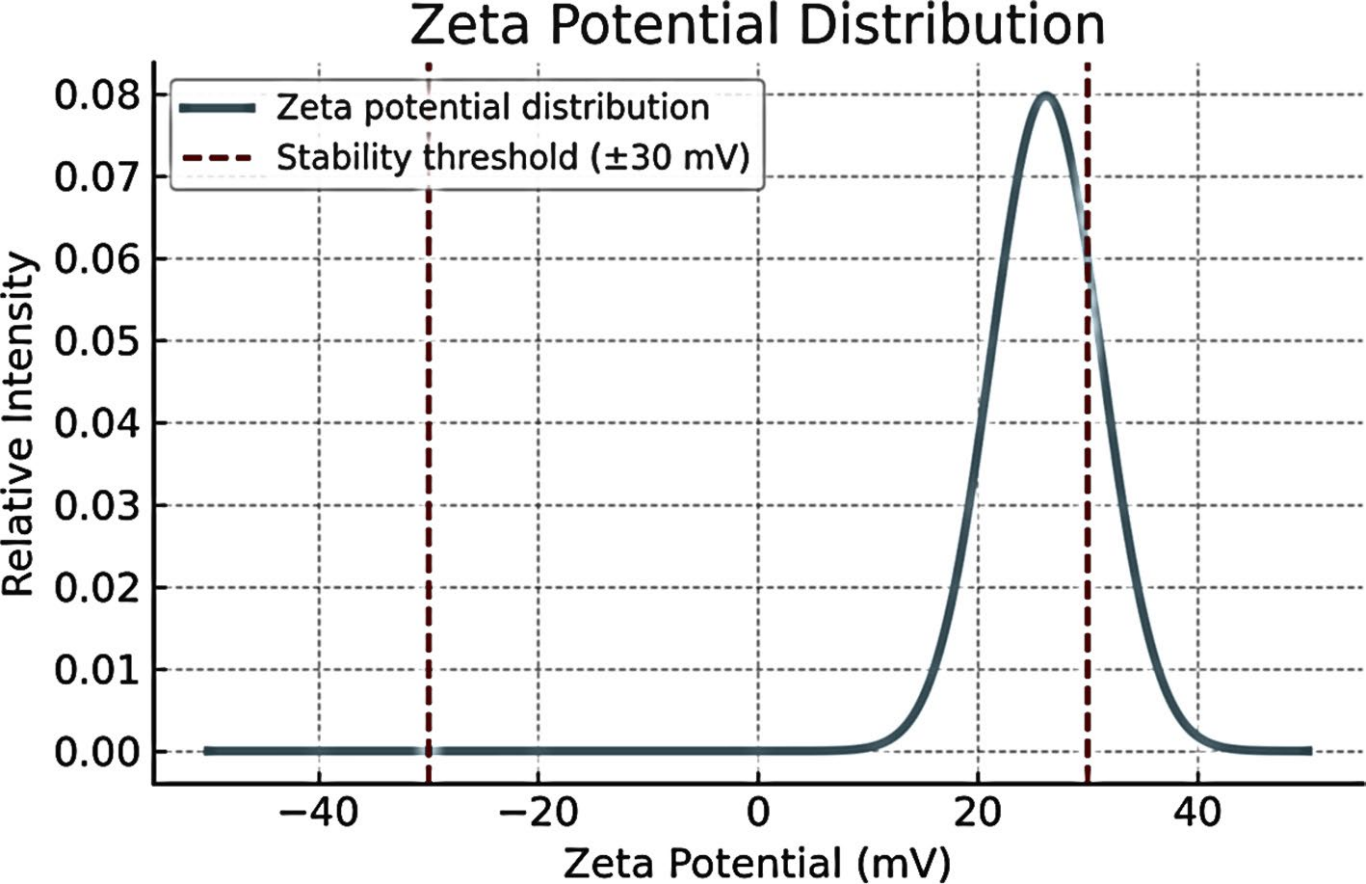
Zeta potential distribution of the nanoparticles showing a sharp peak centered at + 26.2 mV, which is close to the 31.2 mV stability threshold (red dashed lines)

**Fig. 3:**
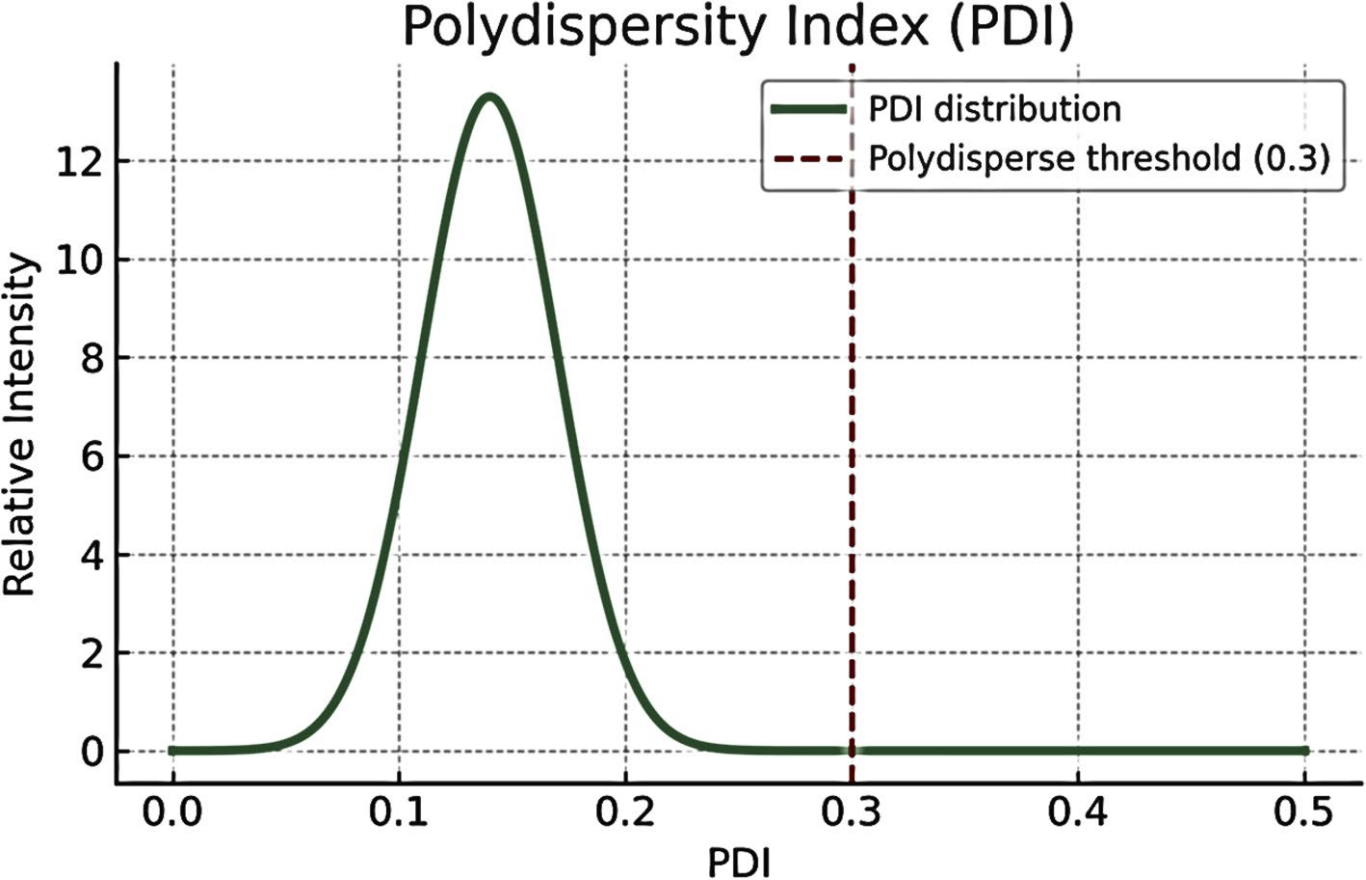
Poly dispersity index (PDI) distribution of the prepared nanoparticles. The obtained PDI value (0.14) lies well below the polydisperse threshold (0.3), confirming a narrow size distribution and high formulation stability

**Fig. 4:**
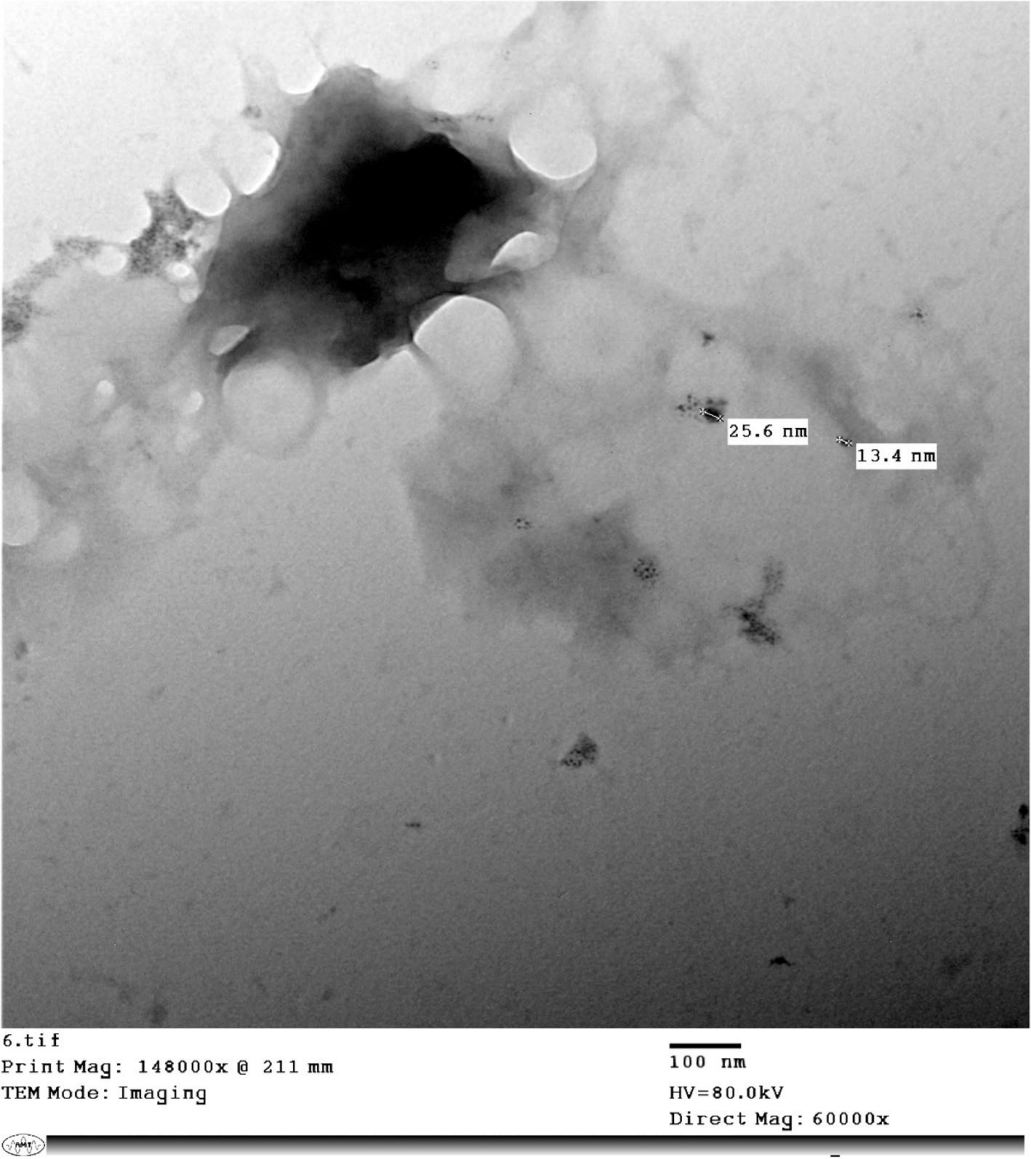
The transmission electron microscopy (TEM) image indicates that the majority of the particles are spherical and range in size from approximately 13.4 to 25.6 nm. the majority of the particles were well-dispersed or isolated, with a few larger agglomerates. the measured size of the formulation falls within the expected moderate range and is in good agreement with the number-based DLS median (D50 ≈ 25.3 nm), supporting a nanosized and monodisperse group content of this formulation

Transmission Electron Microscopy (TEM) images showed predominantly spherical NPs with diameters of approximately 13–26 nm, consistent with the number-based DLS median (D50 ≈ 25.3 nm) (Fig. 1). The presence of some large agglomerates that may explain the high Z-average and volume-weighted mean observed by DLS was also detected despite a PDI ≈ 0.14 (Fig. 2 and Fig. 3). These results suggest that the majority of the formed nanoparticles are in the 20–30 nm size range with low dispersity and a very small fraction of larger aggregates (Fig. 4).

### The GC–MS analysis

**Fig. 5:**
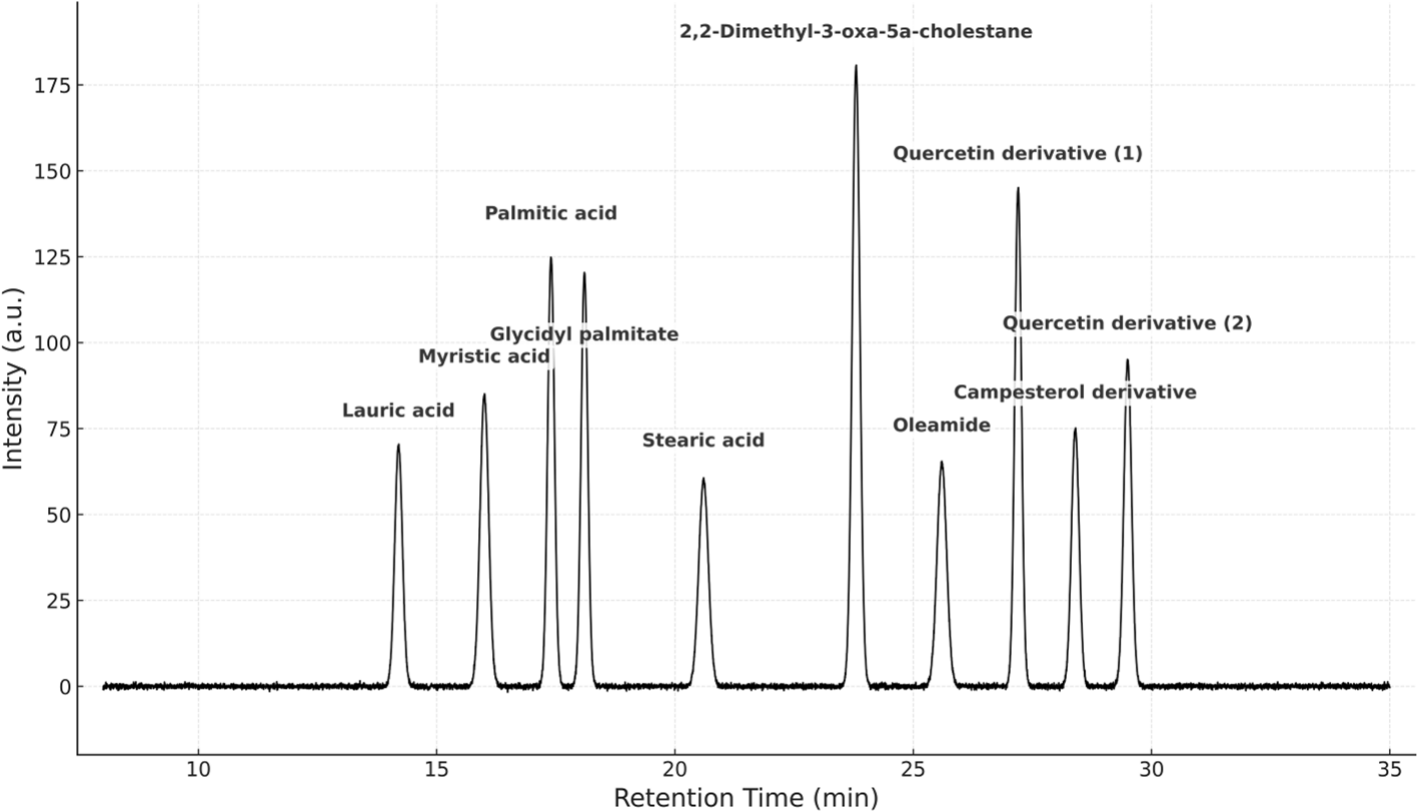
GC–MS chromatogram of APS-NE showing sharp and well-resolved peaks corresponding to the major identified phytoconstituents. the main compounds detected include lauric acid (14.2 min), myristic acid (16.0 min), Palmitic acid (17.4 min), glycidyl palmitate (18.1 min), stearic acid (20.6 min), 2,2-dimethyl-3-oxa-5a-cholestane (23.8 min), oleamide (25.6 min), quercetin derivative (1) (27.2 min), campesterol derivative (28.4 min), and quercetin derivative (2) (29.5 min). The pointed peak morphology indicates a high analytical resolution and confirms the phytochemical richness of APS-NE

As shown in Fig. 5, GC–MS analysis of the tested sample identified several major compounds, quantified by their area percentages. The predominant constituents include fatty acids, steroids, flavonoids, and other bioactive molecules. The sample was particularly rich in fatty acids and their derivatives, with palmitic acid (11.48%) and glycidyl palmitate (11.44%) comprising substantial proportions, indicating a lipid-based origin and potential anti-inflammatory properties. The most abundant compound detected was 2,2-dimethyl-3-oxa-5a-cholestane (17.21%), a steroidal derivative with possible biological activity. Flavonoid derivatives, such as quercetin-based compounds (13.98% and 6.17%), suggest a pronounced antioxidant capacity. Additional identified components include oleamide, a neuroactive fatty acid amide, and various phenolic antioxidants, underscoring the sample’s potential for pharmaceutical or nutraceutical applications.

### MTT assay of APS-NE

**Fig. 6:**
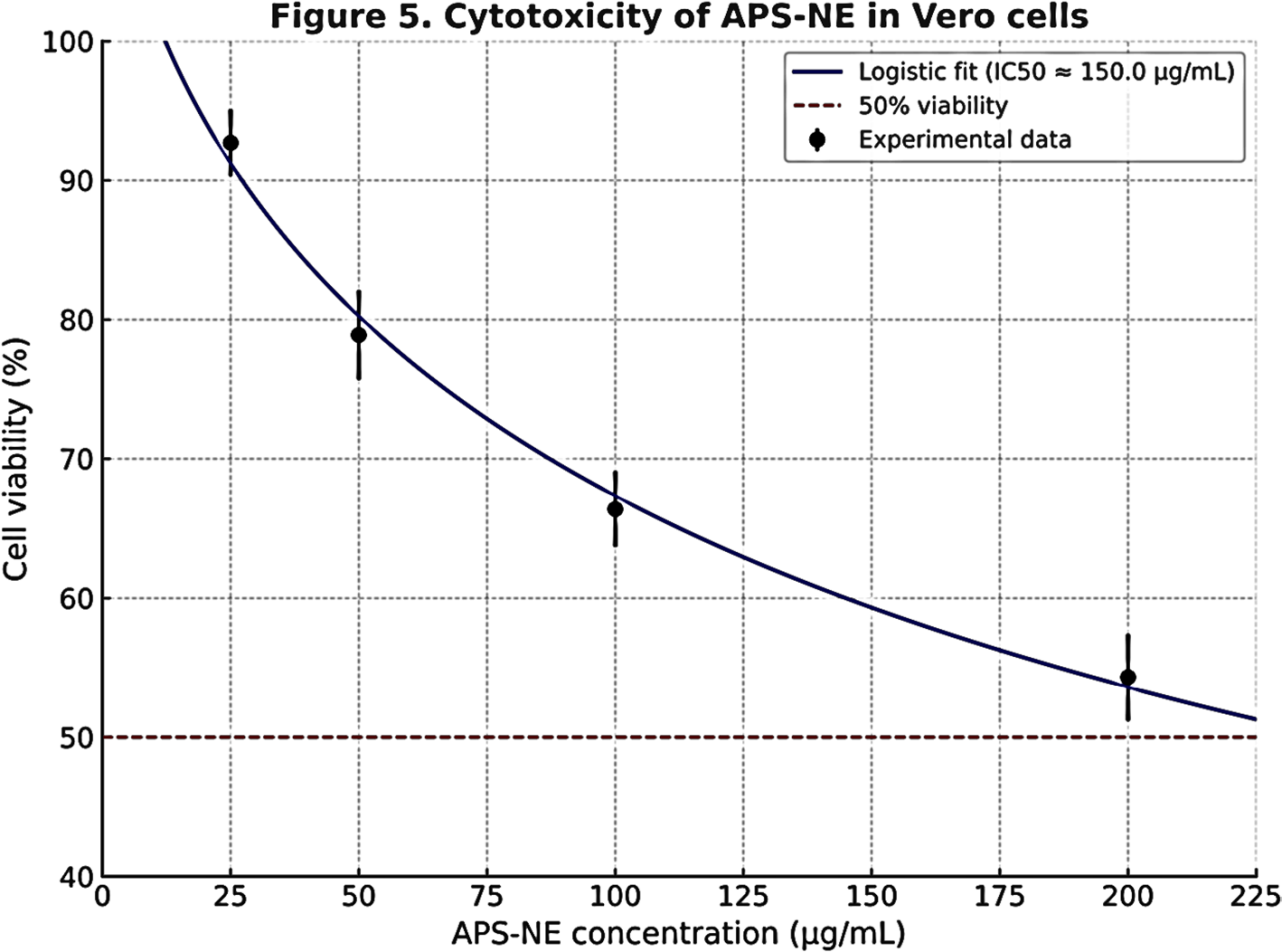
MTT assay for the cytotoxicity of APS-NE to vero cells. values are presented as mean ± SD (n = 3 independent experiments with triplicate wells). IC₅₀ is represented by the dotted line. The IC₅₀ was determined to be 222.1 µg/mL, and the maximum concentration used (200 µg/mL) reduced the viability to 54.3 ± 3.1%

The in vitro cytotoxicity profile of APS-NE in Vero cells (Fig. 6) exhibited a significant dose-dependent decrease in cell viability. The cells still exhibited > 65% viability at 25–100 µg/mL and only 54.3 ± 3.1% at 200 µg/mL. Nonlinear logistic regression fitted an IC₅₀ of approximately 222.1 µg/mL; however, it should be emphasized that the 50% level was not experimentally obtained throughout the concentration series (IC₅₀ > 200 µg/mL by direct count). This result suggests that APS-NE has low cytotoxicity, as it retains more than half of the viable cell numbers at high concentrations. These safe margins confirm the potential for additional biological research.

### Determination of LD50 of the tested drug

**Fig. 7:**
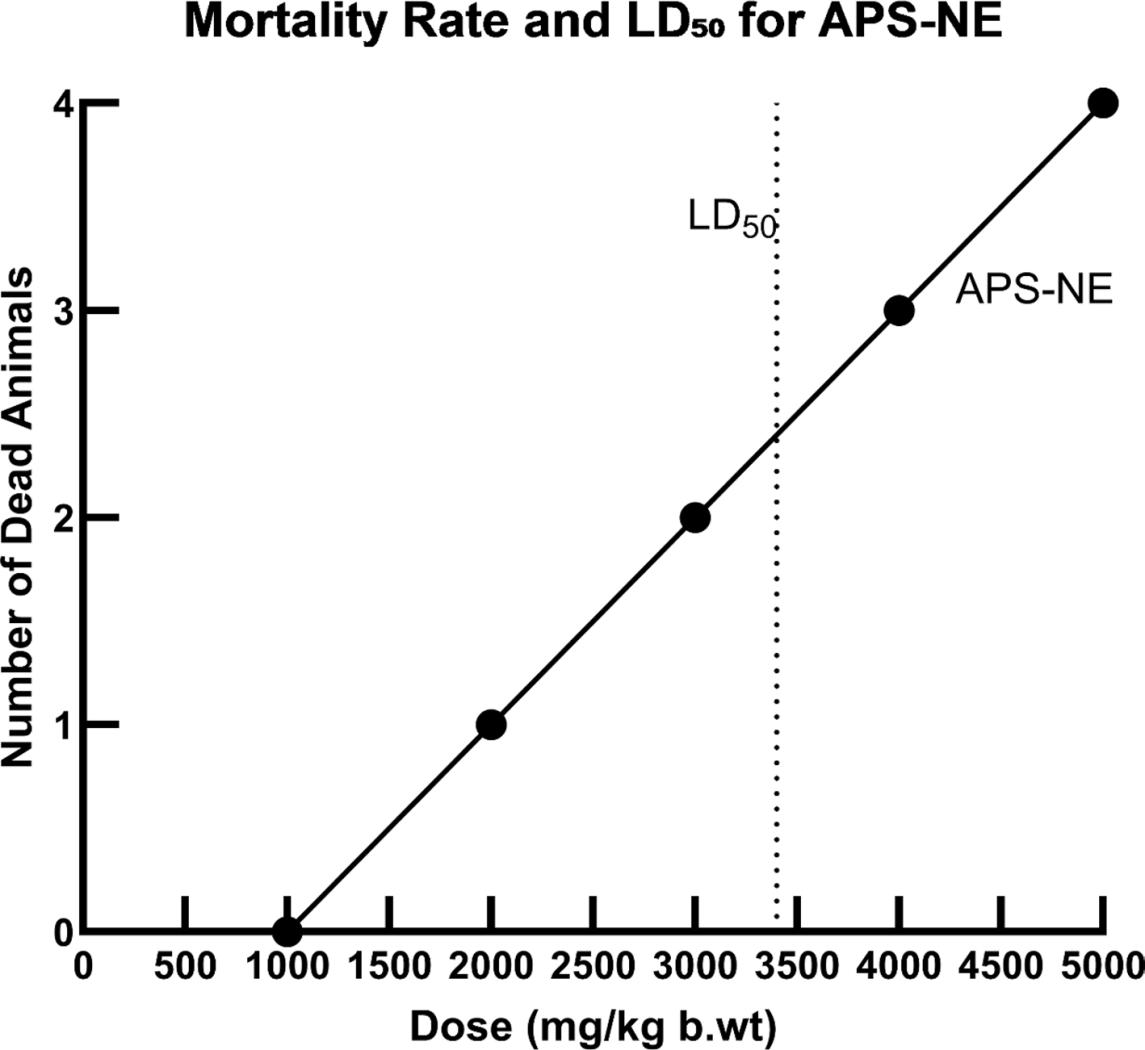
Dose-dependent mortality in mice following oral administration of APS-NE. The LD₅₀ was calculated to be 3400 mg/kg b.wt

The revised dose–response analysis at higher concentrations, as shown in Fig. 7, demonstrates an increased LD₅₀ value of 3400 mg/kg body weight, indicating that APS nanoparticles exhibit low acute toxicity. According to the Hodge and Sterner toxicity scale, substances with LD50 values exceeding 2000 mg/kg are classified as practically non-toxic. These results further substantiate the favorable safety profile of APS-NE and support its suitability for oral administration with minimal risk at therapeutic doses.

### Bioefficacy against *H. dromedarii* life stages

**Fig. 8:**
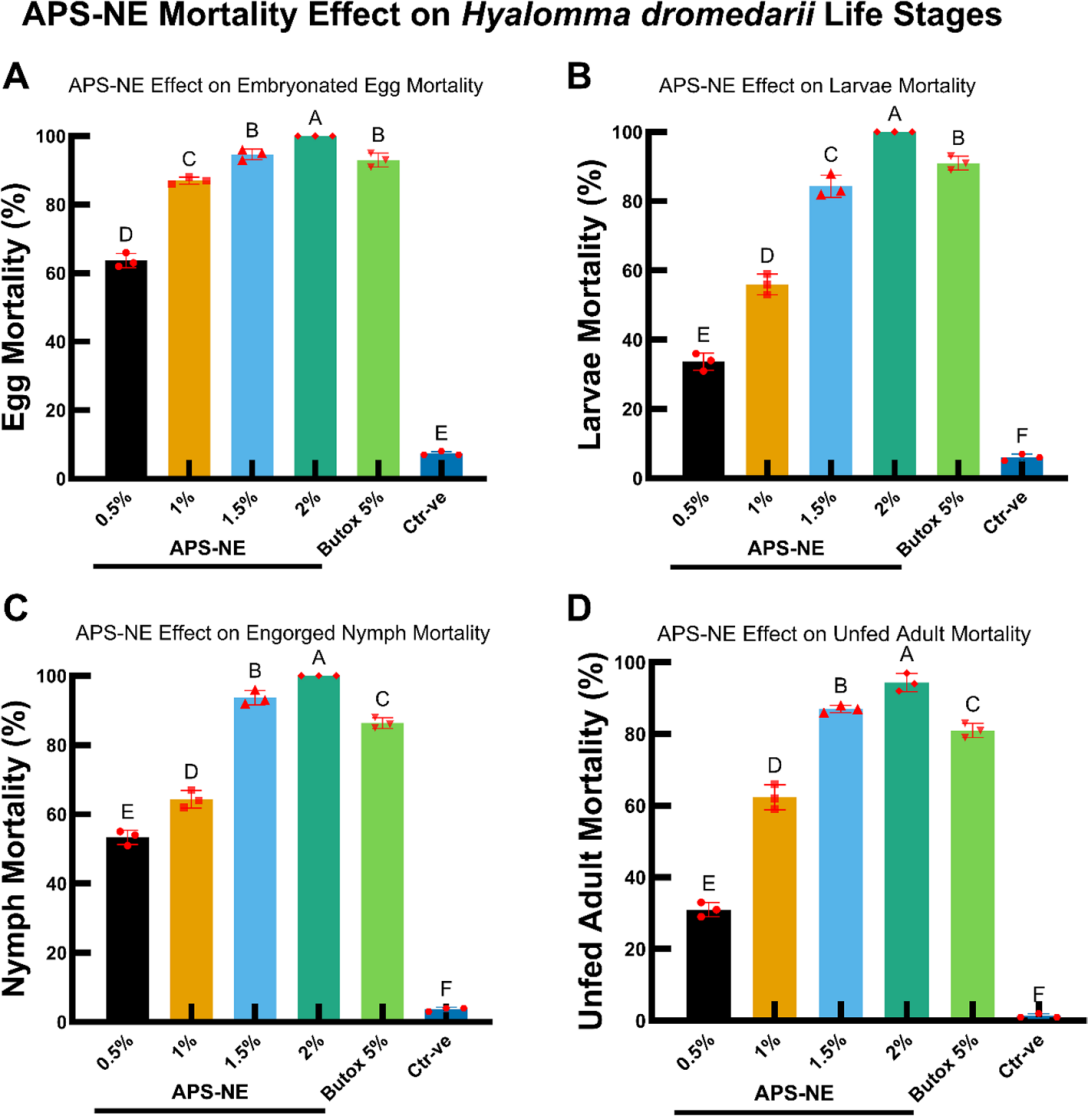
APS-NE mortality effect on different life stages of *H. dromedarii*. bar graphs showing the mortality percentages of different life stages of *H. dromedarii* treated with varying concentrations of APS-NE and Butox 5%: (**A**) embryonated egg mortality, (**B**) larvae mortality, (**C**) engorged nymph mortality, (**D**) unfed adult mortality. different letters above the bars indicate statistically significant differences (p < 0.05) among treatments. error bars represent the standard deviation of the mean (SD)

The biological activity of APS-NE was systematically evaluated at various developmental stages of *H. dromedarii*. Figure 8 displays the mortality percentages for embryonated eggs, larvae, engorged nymphs, and unfed adults after exposure to different concentrations of APS-NE, with Butox 5% serving as a reference. APS-NE induced a concentration-dependent increase in mortality across all stages, with statistically significant differences between the treatment groups (p < 0.05) (Tables S1-S4).

### Impact on reproductive parameters

**Fig. 9:**
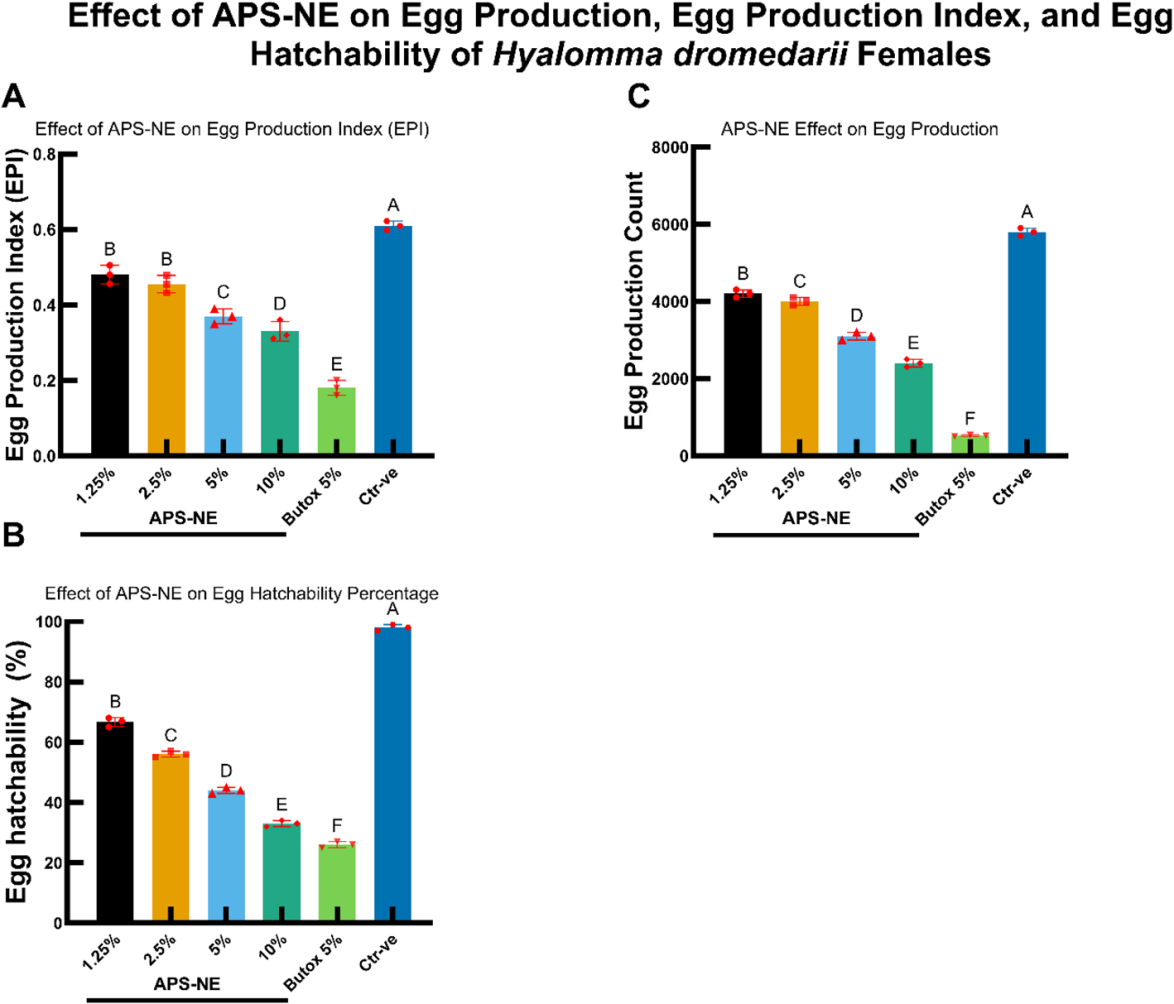
Effect of APS-NE on egg production, egg production index, and egg hatchability of *H. dromedarii* females bar graphs showing the impact of different concentrations of APS-NE and butox 5% on reproductive parameters of *H. dromedarii* females: (**A**) egg production index (EPI), (**B**) egg production count, and (**C**) egg hatchability percentage. different letters above the bars indicate statistically significant differences (p < 0.05) among treatments. error bars represent the standard deviation of the mean (SD)

In addition to causing direct mortality, APS-NE exposure significantly impaired the reproductive parameters of *H. dromedarii* females (Fig. 9). Increasing APS-NE concentrations led to marked reductions in the EPI, total egg count, and egg hatchability percentage (p < 0.05) (Tables S5-S7). These inhibitory effects were evident at all tested concentrations, with the most severe reductions in reproductive output observed at the highest APS-NE doses.

### Dose-dependent mortality and population suppression of *H. dromedarii* by APS-NE

**Fig. 10:**
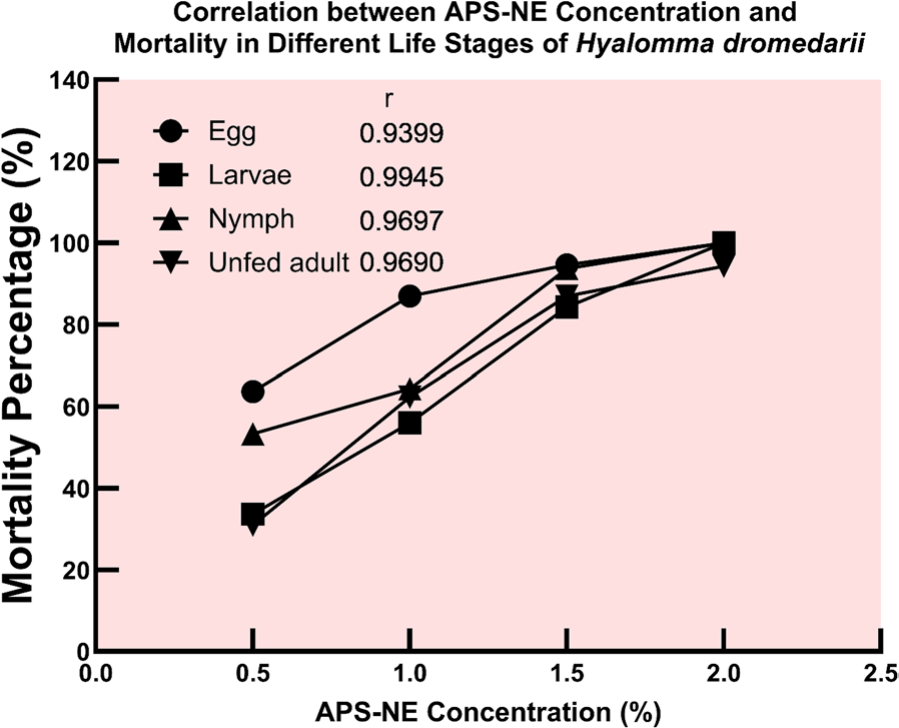
Correlation between APS-NE concentration and mortality percentage in different life stages of *H. dromedarii*. the graph shows the relationship between APS-NE concentration (%) and mortality percentage (%) in four life stages of *H. dromedarii*: egg, larvae, nymph, and unfed adult. Each line represents mortality trends at increasing nanoparticle concentrations (0.5%, 1.0%, 1.5%, 2.0%). Correlation coefficients (r) indicated strong positive correlations between APS-NE concentration and mortality in all stages, with larvae exhibiting the highest correlation (r = 0.9945)

APS-NE demonstrated potent, concentration-dependent effects across all major life stages of *H. dromedarii*, impacting both survival and reproductive parameters. Increasing APS-NE concentrations led to consistent elevations in mortality rates among eggs, larvae, nymphs, and adults. The relationship between nanoemulsion concentration and mortality was robust, as indicated by very strong positive correlations (Pearson’s r > 0.94; Spearman’s ρ = 1), with larval mortality showing the highest responsiveness (r = 0.9945) (Fig. 10). It is important to note that although absolute larval mortality percentages (Fig. 7-B) are lower at some APS-NE concentrations, larvae show the highest sensitivity to APS-NE, as indicated by their strong dose-mortality correlation (r = 0.9945) and lower LC50 thresholds compared to other life stages. This sensitivity highlights their increased vulnerability to APS-NE treatment.

**Fig. 11:**
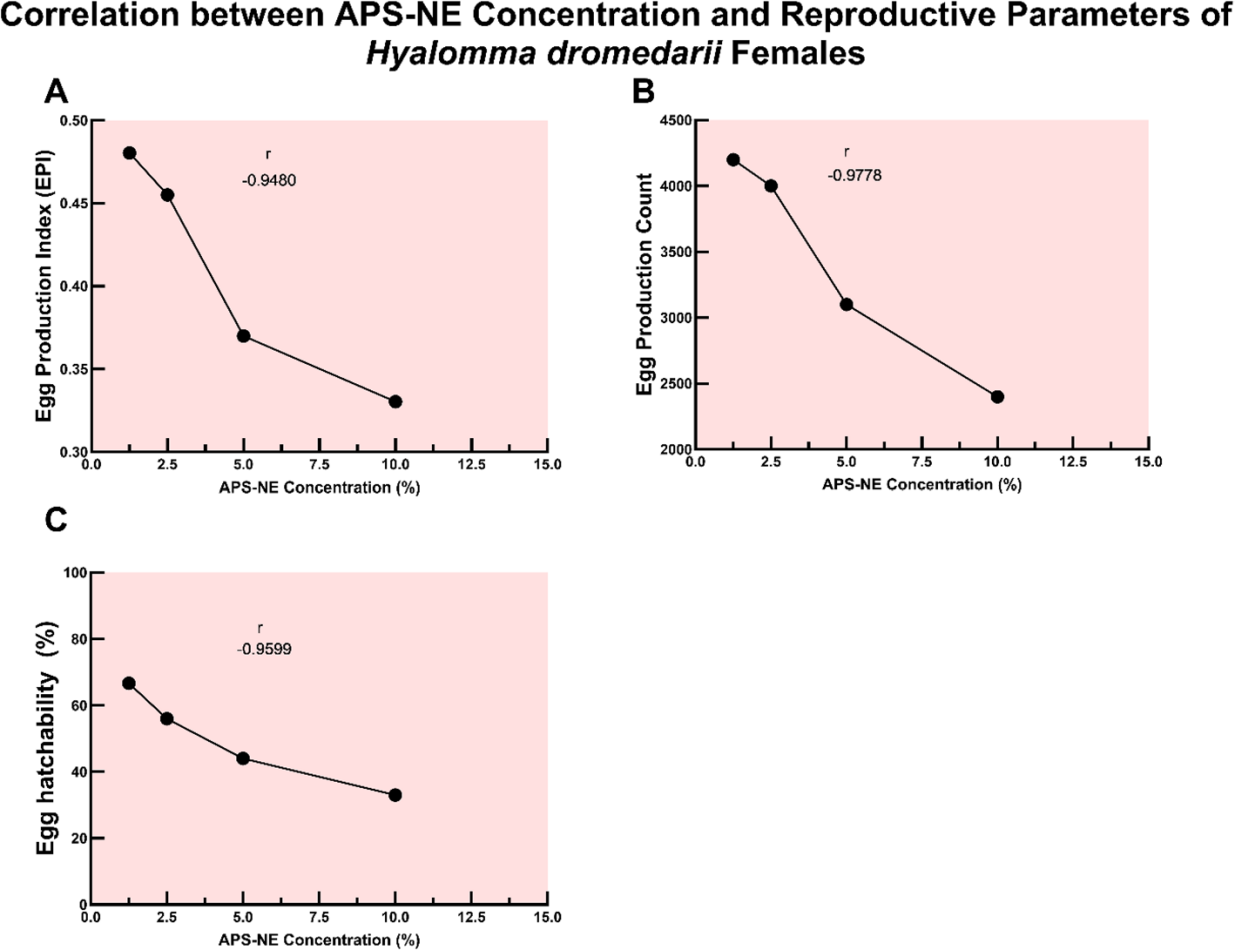
Correlation between APS-NE concentration and reproductive parameters of *H. dromedarii* females. scatter plots showing the negative correlation between APS-NE concentration (%) and reproductive parameters of *H. dromedarii* females: (**A**) egg production index (EPI), (**B**) egg production count, and (**C**) egg hatchability (%). pearson correlation coefficients (r) are indicated in each plot, demonstrating a strong inverse relationship between APS-NE concentration and each reproductive parameter

Concurrently, higher APS-NE concentrations were associated with pronounced reductions in reproductive success. Measures such as the EPI, total egg output, and hatchability all declined markedly with increasing nanoemulsion concentrations. These negative correlations were substantial across all reproductive metrics, underscoring the capacity of APS-NE to disrupt population sustainability (Fig. 11).

**Fig. 12:**
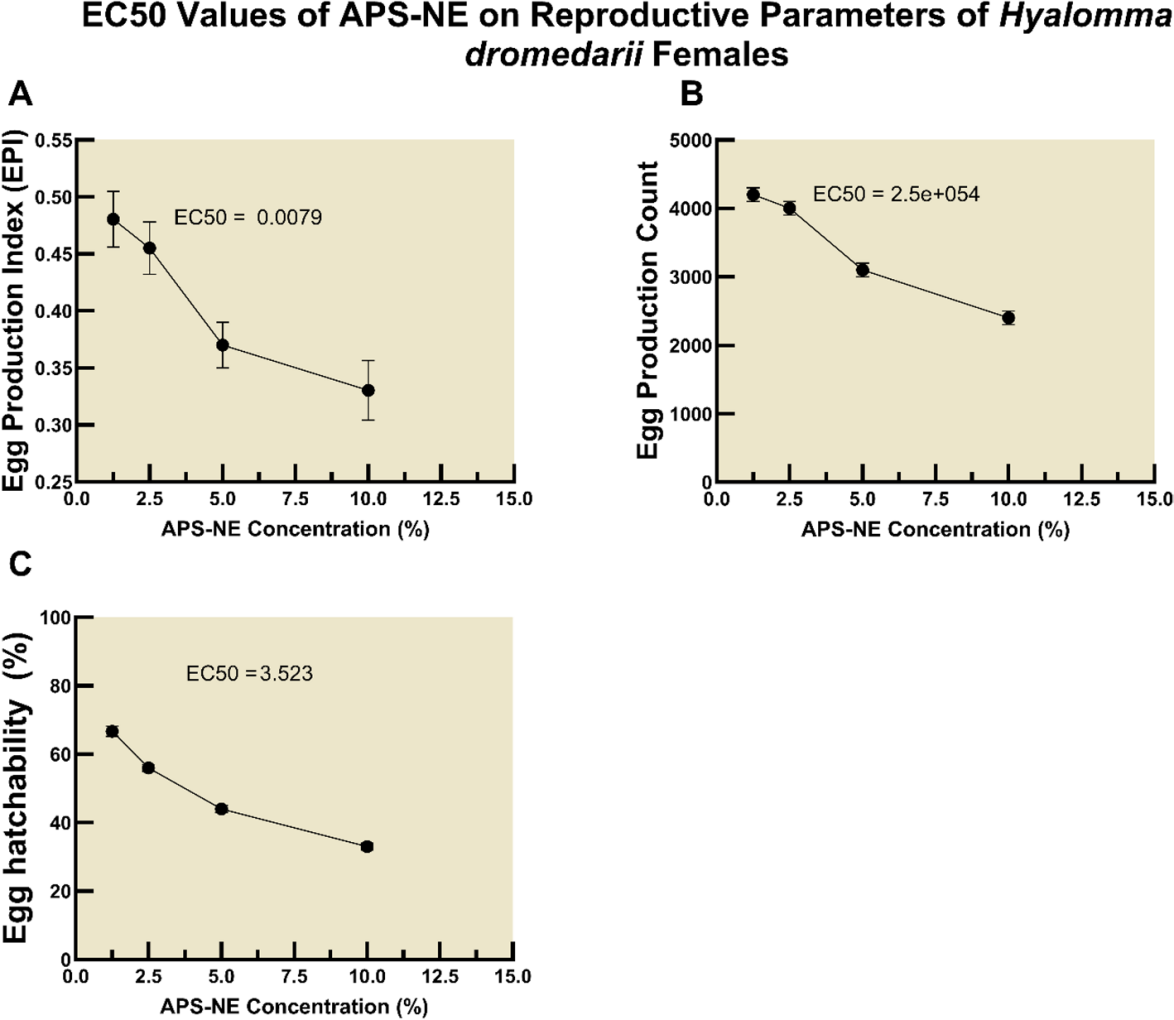
EC50 values of APS-NE on reproductive parameters of *H. dromedarii* females dose–response curves showing the effect of increasing APS-NE concentrations on the reproductive parameters of *H. dromedarii* females. (**A**) egg production index (EPI), (**B**) egg production count, and (**C**) egg hatchability percentage. The EC50 values indicate the concentration (%) of APS-NE required to reduce each reproductive parameter by 50%

Quantitative analysis of dose–response curves further revealed stage-specific sensitivities to APS-NE exposure. The concentrations required to achieve 50% mortality (LC50) varied among developmental stages, with larvae and unfed adults exhibiting lower LC50 values compared to eggs and nymphs, indicating greater vulnerability (Table 1). The analysis determined that the LC50 is at least 15.81 (one-sided 95% lower confidence limit is 5.07) for egg, 4.187 (one-sided 95% lower confidence limit is 1.147) for larvae, 7.627 (one-sided 95% lower confidence limit is 2.428) for nymph, 4.191 (one-sided 95% lower confidence limit is 1.197) for unfed adult. Confidence intervals are 95% profile likelihood intervals. One sided interval (lower bound only) occurs when the upper bound is not identifiable (infinite) from the data at 95% confidence. Upper bounds can be non estimable when the top/bottom plateaus are weakly identified or when data do not fully capture the transition region. Similarly, EC50 determinations for reproductive inhibition confirmed the effectiveness of APS-NE in reducing EPI, egg output, and hatchability by half at defined concentrations (Fig. 12).

**Table 1:** LC50 values of APS-NE for different tick stages of *H. dromedarii*

Tick stage	LC50 (%)
Egg	15.81
Larvae	4.187
Nymph	7.627
Unfed adult	4.191

Collectively, these findings establish APS-NE as a highly effective agent against *H. dromedarii*, exerting direct lethality and significant reproductive disruption in a dose-dependent manner. The consistency of the statistical associations and the clear definition of LC50 and EC50 thresholds reinforce the reliability and efficacy of APS-NE as a promising candidate for integrated tick management strategies.

## Discussion

The present study demonstrated that APS-NE exerted potent acaricidal activity against all developmental stages of *H. dromedarii*. The APS-NE displayed an average particle size of 59.10 ± 0.20 nm and a zeta potential of + 31.2 ± 0.01 mV, indicating high colloidal stability. These results are consistent with previous reports demonstrating similar particle sizes and positive zeta potentials for APS nanoparticles, which contribute to improved stability and bioavailability (Fayez et al. 2025). TEM revealed that the APS nanoparticles were predominantly spherical to sub-spherical, with diameters ranging from 13.4–25.6 nm. The uniform morphology and absence of aggregation indicate successful nanoemulsion formation, corroborating earlier findings on APS-based nanoformulations. Gas chromatography–mass spectrometry analysis identified several bioactive constituents in the APS-NE, including palmitic acid, glycidyl palmitate, and quercetin derivatives. These compounds possess established anti-inflammatory and antioxidant activities, which may act synergistically to enhance the therapeutic efficacy of nanoemulsions (Mahadev et al. 2022).

The results of the MTT assay demonstrate a concentration-dependent cytotoxic effect of APS-NE on the viability of the tested cell line. Cell viability progressively decreased from 100% at 0 µg/mL to approximately 63.8% at 100 µg/mL, with an estimated IC₅₀ value of 222.1 µg/mL. These findings indicate that APS-NE exhibits moderate cytotoxicity, consistent with previous reports on the bioactivity of *Astragalus*-derived polysaccharides, particularly when delivered via nanoformulations to enhance cellular uptake (Yang et al. 2024). Complementary in vivo acute toxicity testing in mice yielded an LD₅₀ value of 3400 mg/kg body weight, classifying APS-NE as practically non-toxic, according to standard toxicity scales. This relatively high IC₅₀ value suggests that the formulation is not overtly toxic at therapeutic concentrations, supporting its potential for safe application in biomedical contexts, such as immunomodulation or anticancer therapy. Nevertheless, further studies, including mechanistic investigations and long-term toxicity assessments, are required to fully characterize the therapeutic index and safety profile of this nanomaterial.

Acute toxicity evaluation using Miller and Tainter method confirmed a dose-dependent increase in mortality, with an LD₅₀ of 3400 mg/kg body weight. According to the Hodge and Sterner (1949) classification, APS-NE is classified as a practically non-toxic compound, indicating a wide safety margin. The high LD₅₀ value supports the tolerability of APS-NE in vivo and its potential suitability for oral therapeutic formulations. These findings align with previous studies demonstrating that nanoemulsion-based delivery systems often possess enhanced biocompatibility and reduced toxicity, attributed to the improved solubility and bioavailability of active constituents (Jacob et al. 2024; Chatzidaki and Mitsou 2025). While the acute toxicity profile is favorable, a comprehensive safety assessment requires further investigation into chronic toxicity, immunotoxicity, and organ-specific effects following repeated or prolonged administration.

Regarding acaricidal activity, APS-NE induced a significant, concentration-dependent increase in mortality across all life stages of *H. dromedarii*. Based on the calculated LC50 values, larvae were the most sensitive stage to APS-NE, followed by nymphs, whereas adults and eggs required higher concentrations to achieve comparable mortality. These results corroborate previous research indicating the heightened vulnerability of early tick developmental stages to botanical and nanoformulated acaricides due to less developed protective barriers and higher metabolic rates (Albogami et al. 2025). Similar efficacy has been reported for nanoemulsions derived from *Azadirachta indica A. Juss* and *Eucalyptus globulus Labill.*, which disrupt the cuticular integrity and physiological function of arthropods (Sandhu et al. 2023).

APS-NE also exhibited potent inhibitory effects on tick reproduction. Increases in APS-NE concentration was associated with significant reductions in egg production index, total egg count, and hatchability. Egg-stage testing was performed to quantify ovicidal activity and reproductive suppression, which are endpoints that directly inform reinfestation risk and environmental persistence. Established assays (egg hatch assay) are widely used to assess these outcomes. Our results align with published findings showing that certain acaricides significantly inhibit egg hatch while others have limited ovicidal effects, underscoring the added value of egg endpoints alongside adult/larval efficacy (Khater et al. 2016; Nabil et al. 2023; Radwan et al. 2023). This suggests interference with reproductive physiology, potentially through the disruption of vitellogenesis or endocrine signaling pathways, as reported in other phytochemical studies (Ibrahium et al. 2022). The inverse correlation between APS-NE concentration and reproductive parameters was consistent and statistically significant, underscoring the formulation’s potential to suppress tick population sustainability. Butox 5% served as a reference acaricide and demonstrated effective performance; however, it did not surpass the efficacy observed at the highest APS-NE concentrations in many cases. This observation is particularly relevant given the increasing incidence of resistance to synthetic acaricides, such as deltamethrin, in *H. dromedarii* populations (Mehlhorn et al. 2010). The prevalence of resistance in camel-rearing regions has diminished the long-term effectiveness of conventional chemical control strategies (Makwarela et al. 2025). The nanoemulsion delivery system confers significant advantages. The small droplet size likely enhances penetration through the tick cuticle and increases the bioavailability of active compounds. Nanoemulsions also improve the stability and dispersibility of botanical extracts in aqueous environments, facilitating their practical application under field conditions. These properties are reflected in the robust dose–response relationships observed for both lethality and reproductive inhibition (Khater et al. 2024).

APS-NE’s efficacy across multiple tick life stages, coupled with its pronounced reproductive disruption, supports its candidacy for inclusion in integrated tick management programs. Unlike conventional acaricides that typically target a single life stage, APS-NE offers broader efficacy that could reduce tick populations more effectively and potentially decrease application frequency. Building on the current findings, which demonstrate significant effects on egg hatchability and egg number reduction, future studies could calculate the Drummond control percentage to further quantify the reproductive suppression. By integrating these metrics into a single, sensitive indicator, this approach would provide a comprehensive assessment of population-level impacts, extending beyond adult mortality and the individual reproductive parameters evaluated here. Such analyses could enhance our understanding of the broader ecological implications of these effects. However, environmental factors such as persistence, degradation rates, and cost-effectiveness require assessment in future field trials. Additional studies should also elucidate the molecular mechanisms underlying APS-NE toxicity and determine whether it induces oxidative stress, neurotoxicity, or hormonal disruption in ticks.

Overall, APS-NE demonstrated consistent and potent acaricidal activity across all developmental stages of *H. dromedarii*, with its nanoformulation enhancing both efficacy and stability. Its capacity to disrupt reproduction further reduces the likelihood of reinfestation. These characteristics position APS-NE as a promising, environmentally compatible alternative to conventional synthetic acaricides. Ongoing studies aim to elucidate its precise mode of action and evaluate its potential organ-specific toxicity in animal models.

## Conclusion

The present study developed an APS-NE as a promising platform for the control of *H. dromedarii* ticks infesting camels, targeting multiple developmental stages. Our approach offers the following eight advantages: i) it effectively targets larvae, nymphs, and adults, ii) it demonstrates low toxicity and a wide safety margin, iii) it can be scaled using established nanoemulsion fabrication techniques to enable large-scale production, iv) it induces significant mortality and inhibits reproductive parameters in a dose-dependent manner, v) it leverages uniform nanoparticle size and formulation stability for consistent performance, vi) it incorporates bioactive compounds such as quercetin derivatives and fatty acids that enhance efficacy, vii) it exhibits favorable biocompatibility with minimal systemic toxicity, and viii) it holds promise for integration into existing pest management programs.

Four limitations of the current formulation are: i) further in vivo validation is required to confirm efficacy under field conditions, ii) the precise pharmacological safety profile in different camel populations remains to be fully characterized, iii) while laboratory results indicate manageable cytotoxicity, long-term safety data are still needed, and iv) the formulation currently requires direct application to infested animals, emphasizing the need for user-friendly delivery systems to facilitate broader adoption.

Given its simplicity, scalability, and dual action on tick survival and reproduction, we envision APS-NE as a valuable candidate for integrated pest management in camel husbandry. Its modular formulation allows for adaptation to other tick species or ectoparasites by modifying the active compound composition. The reconfigurable nature of this nanoemulsion platform positions it as a versatile tool for the rapid deployment of acaricidal interventions in response to emerging veterinary challenges.

## Supplementary Information

Below is the link to the electronic supplementary material.

Supplementary file1 (DOCX 38 KB)

## Data Availability

The data used and analyzed during the current study are available from the corresponding author upon reasonable request. All data generated or analyzed during this study are included in this published article.
