# Supplementary material for: Acaricidal activity of Astragalus polysaccharides nanoemulsion against camel tick, Hyalomma dromedarii
Source: Exp Appl Acarol. 2025 Dec 2;95(4):63. doi: 10.1007/s10493-025-01085-9 (PMC12672669; doi:10.1007/s10493-025-01085-9)
Supplement: Supplementary file 1 — Supplementary file1 (DOCX 38 KB) [file 10493_2025_1085_MOESM1_ESM.docx]

**Supplementary Information**

**For**

**Acaricidal Activity of *Astragalus* Polysaccharides Nanoemulsion Against Camel Tick, *Hyalomma dromedarii***

**Shimaa Abdel-Radi^1^, Mai A. Salem^1^, Fady Sayed Youssef^2^, Mohamed S. Kamel^3^, Mohamed M. El-Bahy^1^ and Reem M. Ramadan^1^**

^1^Department of Parasitology, Faculty of Veterinary Medicine, Cairo University, 12211 Giza, Egypt

^2^Department of Pharmacology, Faculty of Veterinary Medicine, Cairo University, 12211 Giza, Egypt

^3^Department of Medicine and Infectious Diseases, Faculty of Veterinary Medicine, Cairo University, 12211, Giza, Egypt.

***Correspondence**: Reem M. Ramadan, Department of Parasitology, Faculty of Veterinary Medicine, Cairo University

E-mail: [reem.montaser@cu.edu.eg](mailto:reem.montaser@cu.edu.eg)

**Table S1.** One-way ANOVA results with Fisher’s LSD pairwise comparisons for *Hyalomma dromedarii* egg groups treated with APS-NE and Butox 5%. Significance codes are as follows: ns = not significant, *** = p < 0.001, **** = p < 0.0001.

| Comparison | Mean 1 | Mean 2 | Mean Diff. | 95% CI | p-value | Significance |
| --- | --- | --- | --- | --- | --- | --- |
| 0.5% vs 1% | 63.67 | 87.00 | -23.33 | -25.85, -20.82 | <0.0001 | **** |
| 0.5% vs 1.5% | 63.67 | 94.67 | -31.00 | -33.52, -28.48 | <0.0001 | **** |
| 0.5% vs 2% | 63.67 | 100.00 | -36.33 | -38.85, -33.82 | <0.0001 | **** |
| 0.5% vs Butox 5% | 63.67 | 93.00 | -29.33 | -31.85, -26.82 | <0.0001 | **** |
| 0.5% vs Ctr-ve | 63.67 | 7.33 | 56.33 | 53.82, 58.85 | <0.0001 | **** |
| 1% vs 1.5% | 87.00 | 94.67 | -7.67 | -10.18, -5.15 | <0.0001 | **** |
| 1% vs 2% | 87.00 | 100.00 | -13.00 | -15.52, -10.48 | <0.0001 | **** |
| 1% vs Butox 5% | 87.00 | 93.00 | -6.00 | -8.52, -3.48 | 0.0002 | *** |
| 1% vs Ctr-ve | 87.00 | 7.33 | 79.67 | 77.15, 82.18 | <0.0001 | **** |
| 1.5% vs 2% | 94.67 | 100.00 | -5.33 | -7.85, -2.82 | 0.0006 | *** |
| 1.5% vs Butox 5% | 94.67 | 93.00 | 1.67 | -0.85, 4.18 | 0.1745 | ns |
| 1.5% vs Ctr-ve | 94.67 | 7.33 | 87.33 | 84.82,89.85 | <0.0001 | **** |
| 2% vs Butox 5% | 100.00 | 93.00 | 7.00 | 4.48,9.52 | <0.0001 | **** |
| 2% vs Ctr-ve | 100.00 | 7.33 | 92.67 | 90.15,95.18 | <0.0001 | **** |
| Butox 5% vs Ctr-ve | 93.00 | 7.33 | 85.67 | 83.15,88.18 | <0.0001 | **** |

**Table S2.** One-way ANOVA results with Fisher’s LSD pairwise comparisons for *Hyalomma dromedarii* larvae groups treated with APS-NE and Butox 5%. Significance codes are as follows: ****** = p < 0.01, *** = p < 0.001, **** = p < 0.0001.

| Comparison | Mean 1 | Mean 2 | Mean Diff. | 95% CI | p-value | Significance |
| --- | --- | --- | --- | --- | --- | --- |
| 0.5% vs 1% | 33.67 | 56.00 | -22.33 | -26.36, -18.31 | <0.0001 | **** |
| 0.5% vs 1.5% | 33.67 | 84.33 | -50.67 | -54.69, -46.64 | <0.0001 | **** |
| 0.5% vs 2% | 33.67 | 100.00 | -66.33 | -70.36, -62.31 | <0.0001 | **** |
| 0.5% vs Butox 5% | 33.67 | 91.00 | -57.33 | -61.36, -53.31 | <0.0001 | **** |
| 0.5% vs Ctr-ve | 33.67 | 6.00 | 27.67 | 23.64, 31.69 | <0.0001 | **** |
| 1% vs 1.5% | 56.00 | 84.33 | -28.33 | -32.36, -24.31 | <0.0001 | **** |
| 1% vs 2% | 56.00 | 100.00 | -44.00 | -48.02, -39.98 | <0.0001 | **** |
| 1% vs Butox 5% | 56.00 | 91.00 | -35.00 | -39.02, -30.98 | <0.0001 | **** |
| 1% vs Ctr-ve | 56.00 | 6.00 | 50.00 | 45.98, 54.02 | <0.0001 | **** |
| 1.5% vs 2% | 84.33 | 100.00 | -15.67 | -19.69, -11.64 | <0.0001 | **** |
| 1.5% vs Butox 5% | 84.33 | 91.00 | -6.67 | -10.69, -2.65 | 0.0036 | ** |
| 1.5% vs Ctr-ve | 84.33 | 6.00 | 78.33 | 74.31,82.36 | <0.0001 | **** |
| 2% vs Butox 5% | 100.00 | 91.00 | 9.00 | 4.98,13.02 | 0.0004 | *** |
| 2% vs Ctr-ve | 100.00 | 6.00 | 94.00 | 89.98,98.02 | <0.0001 | **** |
| Butox 5% vs Ctr-ve | 91.00 | 6.00 | 85.00 | 80.98,89.02 | <0.0001 | **** |

**Table S3.** One-way ANOVA results with Fisher’s LSD pairwise comparisons for *Hyalomma dromedarii* nymph groups Ttreated with APS-NE and Butox 5%. Significance codes are as follows: *** = p < 0.001, **** = p < 0.0001.

| Comparison | Mean 1 | Mean 2 | Mean Diff. | 95% CI | p-value | Significance |
| --- | --- | --- | --- | --- | --- | --- |
| 0.5% vs 1% | 53.33 | 64.33 | -11.00 | -14.05, -7.95 | <0.0001 | **** |
| 0.5% vs 1.5% | 53.33 | 93.67 | -40.33 | -43.39, -37.28 | <0.0001 | **** |
| 0.5% vs 2% | 53.33 | 100.00 | -46.67 | -49.72, -43.61 | <0.0001 | **** |
| 0.5% vs Butox 5% | 53.33 | 86.33 | -33.00 | -36.05, -29.95 | <0.0001 | **** |
| 0.5% vs Ctr-ve | 53.33 | 3.67 | 49.67 | 46.61, 52.72 | <0.0001 | **** |
| 1% vs 1.5% | 64.33 | 93.67 | -29.33 | -32.39, -26.28 | <0.0001 | **** |
| 1% vs 2% | 64.33 | 100.00 | -35.67 | -38.72, -32.61 | <0.0001 | **** |
| 1% vs Butox 5% | 64.33 | 86.33 | -22.00 | -25.05, -18.95 | <0.0001 | **** |
| 1% vs Ctr-ve | 64.33 | 3.67 | 60.67 | 57.61, 63.72 | <0.0001 | **** |
| 1.5% vs 2% | 93.67 | 100.00 | -6.33 | -9.39, -3.28 | 0.0007 | *** |
| 1.5% vs Butox 5% | 93.67 | 86.33 | 7.33 | 4.28,10.39 | 0.0002 | *** |
| 1.5% vs Ctr-ve | 93.67 | 3.67 | 90.00 | 86.95,93.05 | <0.0001 | **** |
| 2% vs Butox 5% | 100.00 | 86.33 | 13.67 | 10.61,16.72 | <0.0001 | **** |
| 2% vs Ctr-ve | 100.00 | 3.67 | 96.33 | 93.28,99.39 | <0.0001 | **** |
| Butox 5% vs Ctr-ve | 86.33 | 3.67 | 82.67 | 79.61,85.72 | <0.0001 | **** |

**Table S4.** One-way ANOVA results with Fisher’s LSD pairwise comparisons for unfed *Hyalomma dromedarii* adult groups treated with APS-NE and Butox 5%. Significance codes are as follows: ****** = p < 0.01, **** = p < 0.0001.

| Comparison | Mean 1 | Mean 2 | Mean Diff. | 95% CI | p-value | Significance |
| --- | --- | --- | --- | --- | --- | --- |
| 0.5% vs 1% | 31.00 | 62.33 | -31.33 | -35.18, -27.49 | <0.0001 | **** |
| 0.5% vs 1.5% | 31.00 | 87.00 | -56.00 | -59.84, -52.16 | <0.0001 | **** |
| 0.5% vs 2% | 31.00 | 94.33 | -63.33 | -67.18, -59.49 | <0.0001 | **** |
| 0.5% vs Butox 5% | 31.00 | 81.00 | -50.00 | -53.84, -46.16 | <0.0001 | **** |
| 0.5% vs Ctr-ve | 31.00 | 1.33 | 29.67 | 25.82, 33.51 | <0.0001 | **** |
| 1% vs 1.5% | 62.33 | 87.00 | -24.67 | -28.51, -20.82 | <0.0001 | **** |
| 1% vs 2% | 62.33 | 94.33 | -32.00 | -35.84, -28.16 | <0.0001 | **** |
| 1% vs Butox 5% | 62.33 | 81.00 | -18.67 | -22.51, -14.82 | <0.0001 | **** |
| 1% vs Ctr-ve | 62.33 | 1.33 | 61.00 | 57.16, 64.84 | <0.0001 | **** |
| 1.5% vs 2% | 87.00 | 94.33 | -7.33 | -11.18, -3.49 | 0.0013 | ** |
| 1.5% vs Butox 5% | 87.00 | 81.00 | 6.00 | 2.16,9.84 | 0.0053 | ** |
| 1.5% vs Ctr-ve | 87.00 | 1.33 | 85.67 | 81.82,89.51 | <0.0001 | **** |
| 2% vs Butox 5% | 94.33 | 81.00 | 13.33 | 9.49,17.18 | <0.0001 | **** |
| 2% vs Ctr-ve | 94.33 | 1.33 | 93.00 | 89.16,96.84 | <0.0001 | **** |
| Butox 5% vs Ctr-ve | 81.00 | 1.33 | 79.67 | 75.82,83.51 | <0.0001 | **** |

**Table S5**. One-way ANOVA with Fisher's LSD pairwise comparisons of egg production index in *H. dromedarii* females treated with APS-NE and Butox 5%. Significance codes are as follows: ns = not significant,***** = p < 0.05, ****** = p < 0.01, **** = p < 0.0001.

| Comparison | Mean 1 | Mean 2 | Mean Diff. | 95% CI | p-value | Significance |
| --- | --- | --- | --- | --- | --- | --- |
| 1.25% vs 2.5% | 0.4803 | 0.4550 | 0.02533 | -0.01278, 0.06344 | 0.1732 | ns |
| 1.25% vs 5% | 0.4803 | 0.3700 | 0.1103 | 0.07222, 0.1484 | <0.0001 | **** |
| 1.25% vs 10% | 0.4803 | 0.3303 | 0.1500 | 0.1119, 0.1881 | <0.0001 | **** |
| 1.25% vs Butox 5% | 0.4803 | 0.1800 | 0.3003 | 0.2622, 0.3384 | <0.0001 | **** |
| 1.25% vs Ctr-ve | 0.4803 | 0.6100 | -0.1297 | -0.1678, -0.09156 | <0.0001 | **** |
| 2.5% vs 5% | 0.4550 | 0.3700 | 0.08500 | 0.04689, 0.1231 | 0.0004 | *** |
| 2.5% vs 10% | 0.4550 | 0.3303 | 0.1247 | 0.08656, 0.1628 | <0.0001 | **** |
| 2.5% vs Butox 5% | 0.4550 | 0.1800 | 0.2750 | 0.2369, 0.3131 | <0.0001 | **** |
| 2.5% vs Ctr-ve | 0.4550 | 0.6100 | -0.1550 | -0.1931, -0.1169 | <0.0001 | **** |
| 5% vs 10% | 0.3700 | 0.3303 | 0.03967 | 0.00156, 0.07778 | 0.0426 | * |
| 5% vs Butox 5% | 0.3700 | 0.1800 | 0.19000 | 0.1519, 0.2281 | <0.0001 | **** |
| 5% vs Ctr-ve | 0.3700 | 0.6100 | -0.24000 | -0.2781, -0.2019 | <0.0001 | **** |
| 10% vs Butox 5% | 0.3303 | 0.1800 | 0.15030 | 0.1122, 0.1884 | <0.0001 | **** |
| 10% vs Ctr-ve | 0.3303 | 0.6100 | -0.27970 | -0.3178, -0.2416 | <0.0001 | **** |
| Butox 5% vs Ctr-ve | 0.18000 | 0.61000 | -0.43000 | -0.4681, -0.3919 | <0.0001 | **** |

**Table S6.** One-way ANOVA followed by Fisher's LSD pairwise comparisons of egg production count in *H. dromedarii* females treated with different concentration of APS-NE and Butox 5%. Significance codes are as follows: = p < 0.05, **** = p < 0.0001.

| Comparison | Mean 1 | Mean 2 | Mean Diff. | 95% CI | p-value | Significance |
| --- | --- | --- | --- | --- | --- | --- |
| 1.25% vs 2.5% | 4200 | 4000 | 200 | 36.95, 363.0 | 0.0203 | * |
| 1.25% vs 5% | 4200 | 3100 | 1100 | 937.0, 1263 | <0.0001 | **** |
| 1.25% vs 10% | 4200 | 2400 | 1800 | 1637, 1963 | <0.0001 | **** |
| 1.25% vs Butox 5% | 4200 | 520 | 3680 | 3517, 3843 | <0.0001 | **** |
| 1.25% vs Ctr-ve | 4200 | 5800 | -1600 | -1763, -1437 | <0.0001 | **** |
| 2.5% vs 5% | 4000 | 3100 | 900 | 737.0,1063 | <0.0001 | **** |
| 2.5% vs 10% | 4000 | 2400 | 1600 | 1437,1763 | <0.0001 | **** |
| 2.5% vs Butox 5% | 4000 | 520 | 3480 | 3317,3643 | <0.0001 | **** |
| 2.5% vs Ctr-ve | 4000 | 5800 | -1800 | -1963,-1637 | <0.0001 | **** |
| 5% vs 10% | 3100 | 2400 | 700 | 537.0,863.0 | <0.0001 | **** |
| 5% vs Butox 5% | 3100 | 520 | 2580 | 2417,2743 | <0.0001 | **** |
| 5% vs Ctr-ve | 3100 | 5800 | -2700 | -2863,-2537 | <0.0001 | **** |
| 10% vs Butox 5% | 2400 | 520 | 1880 | 1717,2043 | <0.0001 | **** |
| 10% vs Ctr-ve | 2400 | 5800 | -3400 | -3563,-3237 | <0.0001 | **** |
| Butox 5% vs Ctr-ve | 520 | 5800 | -5280 | -5443,-5117 | <0.0001 | **** |

**Table S7.** One-way ANOVA followed by Fisher's LSD pairwise comparisons of egg hatchability percentage in *H. dromedarii* females treated with different concentration of APS-NE and Butox 5%. Significance codes are as follows: **** = p < 0.0001.

| Comparison | Mean 1 | Mean 2 | Mean Diff. | 95% CI | p-value | Significance |
| --- | --- | --- | --- | --- | --- | --- |
| 1.25% vs 2.5% | 66.67 | 56.00 | 10.67 | 8.70, 12.63 | <0.0001 | **** |
| 1.25% vs 5% | 66.67 | 44.00 | 22.67 | 20.70, 24.63 | <0.0001 | **** |
| 1.25% vs 10% | 66.67 | 33.00 | 33.67 | 31.70, 35.63 | <0.0001 | **** |
| 1.25% vs Butox 5% | 66.67 | 26.00 | 40.67 | 38.70, 42.63 | <0.0001 | **** |
| 1.25% vs Ctr-ve | 66.67 | 98.00 | -31.33 | -33.30, -29.37 | <0.0001 | **** |
| 2.5% vs 5% | 56.00 | 44.00 | 12.00 | 10.03,13.97 | <0.0001 | **** |
| 2.5% vs 10% | 56.00 | 33.00 | 23.00 | 21.03,24.97 | <0.0001 | **** |
| 2.5% vs Butox 5% | 56.00 | 26.00 | 30.00 | 28.03,31.97 | <0.0001 | **** |
| 2.5% vs Ctr-ve | 56.00 | 98.00 | -42.00 | -43.97,-40.03 | <0.0001 | **** |
| 5% vs 10% | 44.00 | 33.00 | 11.00 | 9.03,12.97 | <0.0001 | **** |
| 5% vs Butox 5% | 44.00 | 26.00 | 18.00 | 16.03,19.97 | <0.0001 | **** |
| 5% vs Ctr-ve | 44.00 | 98.00 | -54.00 | -55.97,-52.03 | <0.0001 | **** |
| 10% vs Butox 5% | 33.00 | 26.00 | 7.00 | 5.03,8.97 | <0.0001 | **** |
| 10% vs Ctr-ve | 33.00 | 98.00 | -65.00 | -66.97,-63.03 | <0.0001 | **** |
| Butox 5% vs Ctr-ve | 26.00 | 98.00 | -72.00 | -73.97,-70.03 | <0.0001 | **** |
